# Association Between Low Back Pain and Body Mass Index in Pakistani Population: Analysis of the Software Bank Data

**DOI:** 10.7759/cureus.23645

**Published:** 2022-03-30

**Authors:** Ali Sarfraz Siddiqui, Sidra Javed, Shemila Abbasi, Tanveer Baig, Gauhar Afshan

**Affiliations:** 1 Anaesthesiology, Aga Khan University Hospital, Karachi, PAK; 2 Anesthesiology, Shaheed Mohtarma Benazir Bhutto Institute of Trauma, Karachi, PAK; 3 Anaesthesiology, The Aga Khan University, Karachi, PAK

**Keywords:** public health, lumbar radicular pain, body mass index, obesity, low back pain

## Abstract

Background: Obesity is a growing public health concern and is one of the leading causes of human suffering and disability worldwide. The number of overweight and obese people is dramatically increasing, and local data showed that low back pain (LBP) is more common in people with obesity, prolonged sitting jobs, psychological disorders, and lack of exercise.

Methods: This study was conducted in a cohort of 300 adult patients of either gender who visited a pain management clinic with LBP. Patient data were retrieved from the hospital software program and recorded in a pre-designed proforma. The data included the patient’s age, gender, weight, height, BMI, comorbidities, site of pain, duration of pain, distribution of pain, severity of pain, history of spinal trauma, previous spinal surgery, and working diagnosis.

Results: Out of 300 patients with LBP, 185 (61.7 %) were female and 115 (38.3%) were male, of these, 224 (74.6%) were overweight or obese. One hundred and three (34.3%) had axial back pain and 197 (65.7%) patients had lumbar radicular pain. Linear regression analysis showed that 17% variability in pain scores in both genders can be explained by the increase in BMI. There is a statistically significant relationship, i.e. P=0.0005, exists between pain score and BMI.

Conclusion: This study showed the strong association between obesity and LBP in the Pakistani population. Approximately, 75% were overweight or obese in our LBP population-based cohort and this association was stronger among women than men.

## Introduction

Low back pain (LBP) is a great public health issue and the second most common cause of seeking advice from a physician [1]. It is the leading source of human suffering and disability worldwide and is often associated with mental and behavioral conditions such as depression and anxiety [2]. Approximately 30% of the world community suffers from LBP and 80% report LBP at some point in their lives [3]. It has been shown that 40.65% of the Pakistani population above the age of 50 years suffers from LBP. It is 2.5 times higher among women who never do moderate physical activity in their routine life [4]. Local data also showed that LBP is more common in the Pakistani population with obesity, prolonged sitting jobs, psychological disorders, lack of exercise, lack of health awareness, and heavy lifting jobs. Its prevalence is higher in urban than in rural areas [5].

Obesity is a growing public health concern worldwide. Globally, the number of overweight and obese people is dramatically increasing. The data from the United States national health and nutrition examination survey 2017-2018 showed that the age-adjusted prevalence of obesity is 42.4%, while the age-adjusted prevalence of severe obesity is 9.2% among adult patients, aged 20 and above. The general prevalence of obesity is the same in men and women, however, the prevalence of severe obesity is more in women and adult patients, aged 40-59 years [6]. Pakistan is currently suffering from an emerging epidemic of obesity [7]. In Pakistan, according to World Health Organization (WHO) data, 26% of women and 19% of men are obese and the prevalence is highest in urban areas [8].

Obesity is considered a risk factor for LBP due to underlying biological plausibility. Putting on more weight causes increased mechanical loading on lumbar vertebrae that results in a series of changes [9]. It has been shown that obese patients treated for LBP have better outcomes when they reduce body weight, especially in morbid obesity (BMI 40 kg.m2 and above) [10]. Obesity may have both, biochemical and inflammatory, effects on the spine. It is usually related to disc degeneration as increased body weight might result in wear and tear on different structures of the spine, e.g., discs, joints, and ligaments [11]. The relation between LBP and obesity is debatable. Many reviews suggested that BMI is not a significant predictor of LBP [12].

To date, studies on the association between obesity and LBP in low and middle-income countries are scarce. This study was conducted to determine the association of LBP and BMI in the Pakistani population and the frequency of common LBP conditions among such patients.

## Materials and methods

This study was conducted in a cohort of 300 adult patients who visited the pain management clinic with chronic LBP. Exemption from the Ethics Review Committee of Aga Khan University (ERC# 2018-0426-364) was granted for this study.

Study design: A retrospective observational study

Patient data were retrieved from the hospital software program PMCS (Pain Management Clinic System) and recorded in a pre-designed proforma for a period from 1st January 2017 to 31st May 2018.

Inclusion and exclusion criteria

All adult patients of either gender presenting to the pain management clinic of Aga Khan University Hospital with a history of chronic LBP for more than 3 months were included. Patients with missing data and follow-up patients were excluded from the study. All patients were included in this retrospective observational study by reviewing data in the electronic medical record, PMCS by one of the authors as per the availability. The patients’ medical records were also reviewed for any additional information and all the information were recorded in a pre-designed proforma. The data included the patient’s age, gender, weight, height, BMI, comorbidities, site of pain, duration of pain, distribution of pain, the severity of pain, history of spinal trauma, and previous spinal surgery and working diagnosis. BMI [13] is categorized as; underweight (BMI < 18.5 kg.m^2^), normal weight (BMI 18.5 to 24.9 kg.m^2^), overweight (BMI 25 to 29.9 kg.m^2^), obesity (BMI 30 to 39.9 kg.m^2^), morbid obesity (BMI 40 to 49.9 kg.m^2^), and super obesity 50 to 59.9 kg.m^2^.

Statistical analyses

Statistical analyses were performed using Statistical Packages for Social Science version 19 (SPSS Inc., Chicago, IL). Mean and standard deviation was computed for quantitative variables while frequency and percentage were estimated for qualitative variables. A P-value of ≤ 0.05 was considered significant.

## Results

Three hundred patients were included during the study period. One hundred and eighty-five (61.7 %) patients were female while 115 (38.3%) were male. The mean age of the study patient was 51.24±18.12 years and most of them had hypertension (36.3%) followed by diabetes (30.3%) (Table 1).

**Table 1:** Demographic characteristics and comorbid of patients (n=300) IHD: ischemic heart disease

Variables	Point Estimates
Age (Years)	51.24±18.12
Weight (kg)	73.75±15.91
Height (cm)	158.2±8.75
BMI (kg/m^2^)	29.43±5.93
Gender	
Male	115(38.3%)
Female	185(61.7%)
Comorbid	
Hypertension	109(36.3%)
Diabetic mellitus	91(30.3%)
IHD	16(5.3%)
Hypothyroid	12(4%)
Asthma	7(2.3%)
Others	15(5%)

**Figure 1:**
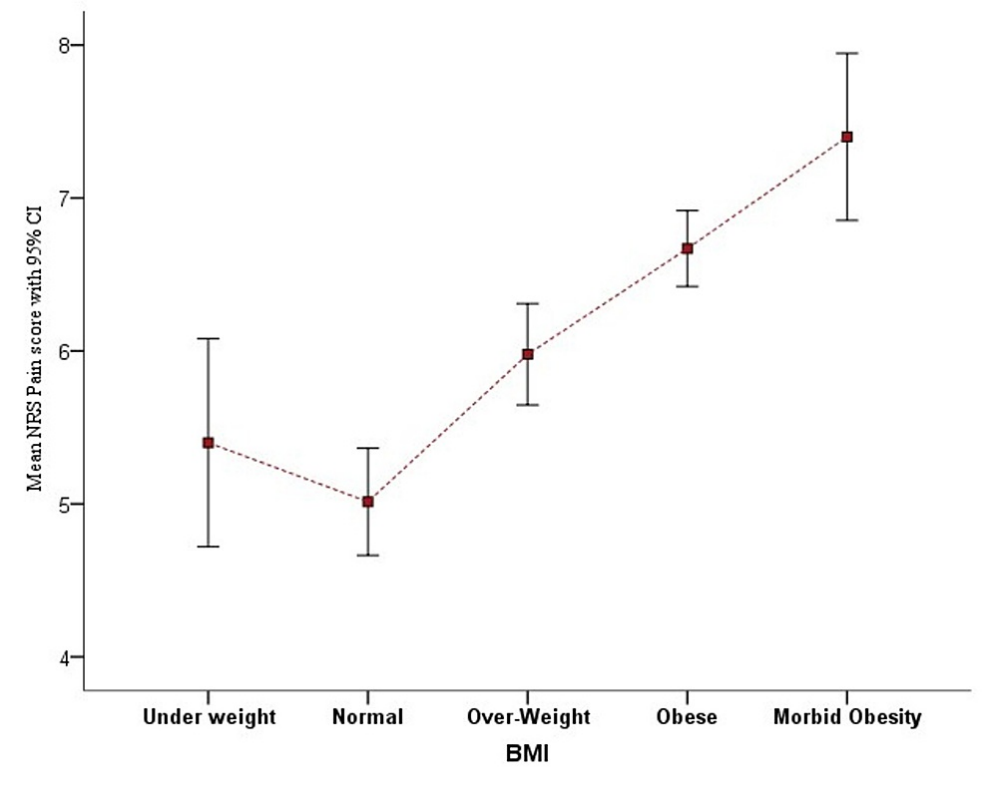
Comparison of mean NRS pain score among BMI categories NRS: numerical rating pain score

Out of 300 study patients, five (1.67%) patients were underweight (BMI < 18.5 kg.m2), 71 (23.67%) had BMI within normal range (18.5 to 24.9 kg.m2), 91 (30.33%) were overweight (BMI 25 to 29.9 kg.m2), 118 (39.33%) were obese (BMI 30 to 39.9 kg.m2), and 15 (5%) were morbidly obese (BMI 40 to 49.9 kg.m2). The difference in mean numerical rating pain score (NRS) scores among the different BMI categories of patients is statistically significant (P=0.0005). The mean NRS pain score of overweight, obese, and normal-weight patients was statistically significant (P=0.001) (Figure 1).

Among 300 chronic LBP patients, 103 (34.3%) patients had axial pain (confined to the low back only), and 197 (65.7%) patients had lumbar radicular pain (pain radiating down the leg). Among patients with radicular pain; 40 (20.3%) patients had pain radiating to the lower limb up to the knee while in 157 (79.7%) patients, pain radiates up to the ankle or foot. Thirty-four (11.3%) patients had a history of spine trauma. Fourteen (4.7%) patients had a history of spine surgery.

**Figure 2:**
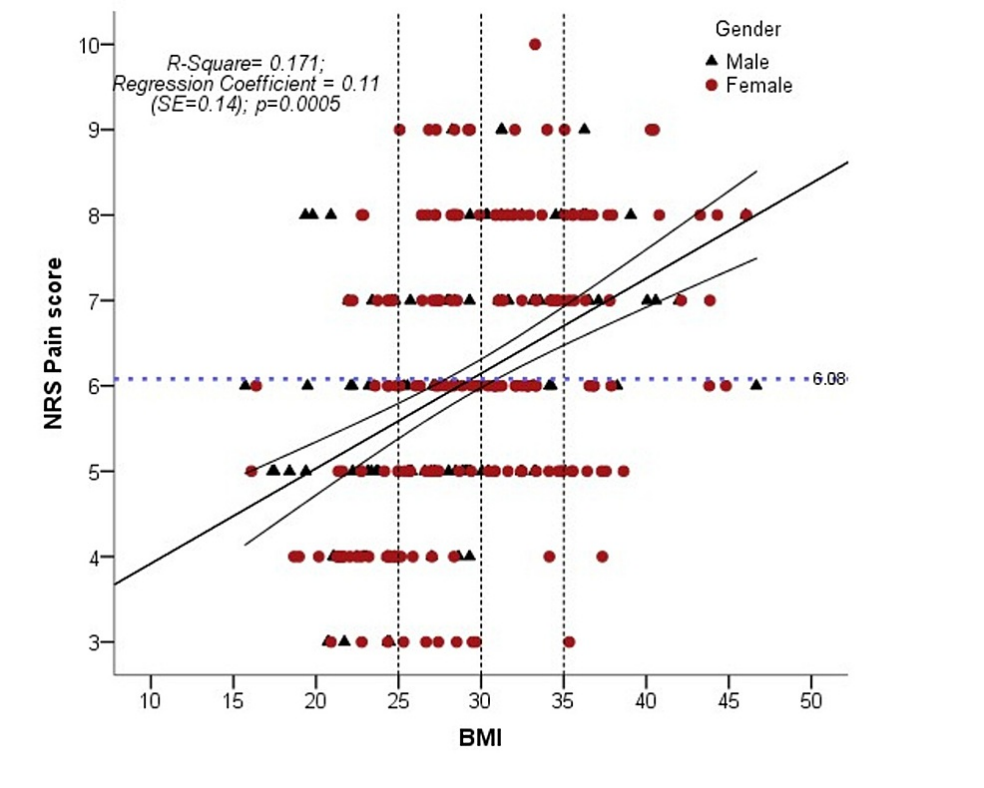
Association of mean pain score and body mass index (n=300) NRS: numerical rating pain score

Subgroup analysis showed that overweight and obese patients are more prone to have lumbar radicular pain, spinal stenosis, disc degenerative disease, and sacroiliac joint pain as compared to normal-weight patients. Ninety-three (31%) patients had a working diagnosis of lumbar radicular pain (Sciatica), 81 (27%) patients had disc degenerative disease, 31 (10.3%) had non-specific back pain, 26 (8.7%) had spinal stenosis, 11 (3.7%) had metastatic cancer pain, 15 (5%) had sacroiliac joint pain, four (1.3%) had failed back surgery syndrome, six (2%) had lumbar facet joint pain while 33 (11%) had miscellaneous causes of back pain (like mixed pain, myofascial pain, discitis). Linear regression analysis shows that 17% variability in NRS pain scores in both genders can be explained by the increase in BMI. Regression coefficient 0.11 shows that with each unit change in BMI there will be an 11% increase in pain score. There is a statistically significant relationship, i.e. P=0.0005, exists between pain score and BMI (Figure 2).

**Figure 3:**
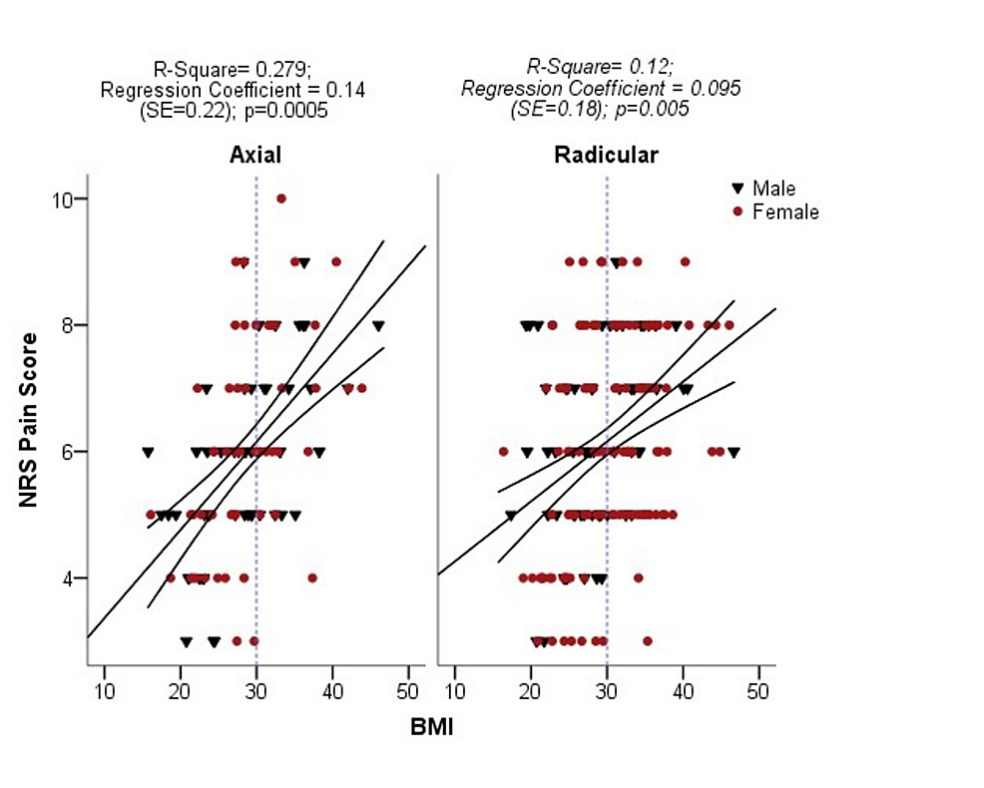
Association of mean pain score and body mass index according to pain distribution (n=300) NRS: numerical rating pain score

A statistically significant relationship (P=0.0005) also exists between pain score and BMI regarding the distribution of pain (axial vs radicular). Analysis showed that 28% variability in NRS pain score is related to the increase in BMI in patients with axial back pain while 12% in patients with lumbar radicular pain (Figure 3).

Results regarding various BMI categories and comorbid conditions of study patients showed that one hundred and sixty-seven (55.66%) patients had one or more disease conditions with chronic LBP. Out of 118 obese patients with LBP, 55 (46.61%) had diabetes mellitus while nine out of 12 hypothyroid patients were overweight or obese.

## Discussion

Among the 300 patients included in this study, data showed a strong association between overweight and obesity with LBP. In the subgroup analysis, we found that 30.33% of LBP patients were overweight, 39.33% were obese, and 5% were morbidly obese. Current literature also showed a significant association of obesity with LBP. A person with a high BMI has two times increased probability of having LBP as compared to an individual with a normal BMI [14]. The relationship between overweight, obesity, and LBP is highlighted in a recent US-based cross-sectional national health survey. Reports showed the odds for LBP within the past 3 months was 1.27 (95% CI: 1.16-1.38) and 1.72 (95% CI: 1.60-1.85) times higher in overweight and obese patients, respectively. In comparison with men, the odds of LBP were significantly higher in women [15]. These findings are similar to the results of the current study.

A recent study mentioned that high BMI is strongly associated with an increased prevalence of LBP [16]. The findings of the current study showed the association of LBP with increasing BMI. These findings are consistent with several studies that have assessed risk factors for LBP. These studies have also reported age, sex, smoking, low income, low education, occupations, physical activity level, and depression, as risk factors [17,18]. They have mainly investigated the association between obesity and LBP and most of them are cross-sectional surveys. These studies support the association between obesity and LBP [19,20].

There are several mechanisms proposed that can explain the relationship between obesity and LBP. First, the increased mechanical load on the back due to obesity causes a higher compressive force on the spine during various physical activities. Second, obesity makes the person vulnerable to have injuries and systemic chronic inflammation. This will lead to increased production of cytokines and activation of pro-inflammatory substances (tumor necrosis factor-alpha (TNF-α), interleukin-6 (IL-6), and blood levels of IL-6) which are elevated in obese patients and causing pain [21]. There is also a stronger association between LBP and abdominal obesity as compared to LBP with generalized obesity [22]. Current literature also shows that smoking and a sedentary lifestyle increase the risk of LBP, however, this information was missing in our pre-designed software database. This is a limitation of our study as these factors are not available and we are co-relating LBP mainly with obesity [23]. The present study also showed that obesity was associated with pain severity (mean pain score on NRS). Obesity as a risk factor for pain severity has been found in previous studies. Current literature suggested that obesity is a risk factor for increased severity of pain and quality of life. However, on the other hand, few studies have not shown the association between the severity of pain and obesity [24].

A previous study showed that the gender difference was less evident for the increasing prevalence of LBP, however, the current study demonstrated an increased incidence of LBP in women [25]. Similar results were also found in a study where female sex and obesity were factors significantly associated with the development of musculoskeletal pain, especially LBP. The gender differences could be due to hormone-related obesity causing altered pain sensitivity. It is biologically plausible that a gender disparity in the pathophysiology of LBP exists in different geographical areas as cultural influences have an impact on daily lifestyle, i.e., diet and physical activities leading to obesity [26]. The current study also found that patients with diabetes mellitus and hypertension have a higher prevalence of LBP and these findings are similar to several earlier studies [27,28].

Many case-control studies have demonstrated a positive relationship between increased BMI and lumbar disc herniation in both genders. Lumbar disc herniation is an important cause of LBP and lumbosacral radicular pain [29]. Subgroup analyses in the current study showed that overweight and obese patients are more prone to have lumbar radicular pain, spinal stenosis, disc degenerative disease, and sacroiliac joint pain as compared to normal-weight patients.

A systematic review has shown that increased body fat is positively related to generalized pain with LBP, knee pain, and foot pain. There is also a positive relationship between increasing body fat and generalized body pain and single-site joint pain in the lower back [30]. Several mechanisms can explain the relationship between body fat and musculoskeletal pain. They include the up-regulation of cytokines secreted by adipose tissue which are referred to as adipokines. Leptin, a pro-inflammatory adipokine predominately expressed by subcutaneous adipose tissue is also associated with generalized body pain in women [31].

Strengths of the study

The current study includes a nationally representative population-based sample of a lower and middle-income country (LMIC). This study may contribute as a reference for future research on LBP and LMIC. The findings of the study have important implications for the prevention and management of LBP in the Pakistani population, however, further research via longitudinal studies needs to be done.

Limitation of the study

The current study has its limitations as it was a single-center study with a relatively small sample. In addition, data was retrieved from the pre-designed software program therefore some of the common risk factors like smoking and the mechanical lifestyle of a person were missed.

## Conclusions

The current study shows the strong association between obesity and LBP in the Pakistani population. Approximately 75% were overweight or obese in our LBP population-based cohort and this association was stronger among women than men. The study highlighted the importance of obesity as it may increase the risk of LBP, lumbar radicular pain, spinal stenosis, disc degenerative disease, and sacroiliac joint pain.
